# Interpreter use in Nordic paediatric oncology care – a multicentre cross-sectional study

**DOI:** 10.2340/1651-226X.2026.46078

**Published:** 2026-09-08

**Authors:** Melissa Jakobsson, Helena Ventovaara, Eva Broström, Pernilla Pergert, Elisabet Tiselius, Johanna Granhagen Jungner

**Affiliations:** a Department of Women’s and Children’s Health, Karolinska Institutet, Stockholm, Sweden; b Karolinska University Hospital, Stockholm, Sweden; c Centre for Research Ethics & Bioethics, Department of Public Health and Caring Sciences, Uppsala University, Uppsala, Sweden; d Department of Swedish Language and Multilingualism, Institute for Interpreting and Translation Studies, Stockholm University, Stockholm, Sweden

**Keywords:** language barriers, paediatric, Nordic countries, survey, questionnaire

## Abstract

**Background and purpose:**

Tailored, language-appropriate information is fundamental for children and their families in paediatric oncology, influencing both the quality of care and clinical outcomes. Yet, for families who speak another language, interpreter services are not used to the extent necessary to ensure equitable and patient-safe communication. Knowledge about how healthcare professionals (HCPs) (registered nurses [RNs] and medical doctors [MDs]) experience and manage interpreter use is limited. To address this gap, this study aimed to explore the obstacles to interpreter use in Nordic paediatric oncology care and to examine the challenges HCPs experience during interpreter‑mediated conversations.

**Participants/materials and methods:**

A cross-sectional multicentre survey study involving 453 RNs and MDs from 20 paediatric oncology centres across the Nordic region. The Communication over Language Barriers Questionnaire was used in each country’s majority language. Descriptive and non-parametric analyses were used to summarise and compare the data between countries and professions. Group differences were tested using Pearson’s chi-square or Fisher–Freeman–Halton exact tests, with *p* < 0.05 considered significant.

**Results:**

Time constraints in acute but also in planned care situations were identified as the most common obstacles to interpreter use. In addition, when using interpreters, the respondents sometimes or often experienced challenges such as uncertainty about the accuracy of the interpretation and about patients’ understanding of the given information.

**Interpretation:**

To ensure equitable, safe, and person‑centred care for children and their families, there is an emergent need for improved communication strategies and a need for better organisational support, easier interpreter access, and stronger systems for quality-assured interpreter services across the Nordic region.

## Graphical abstract

**Figure:**
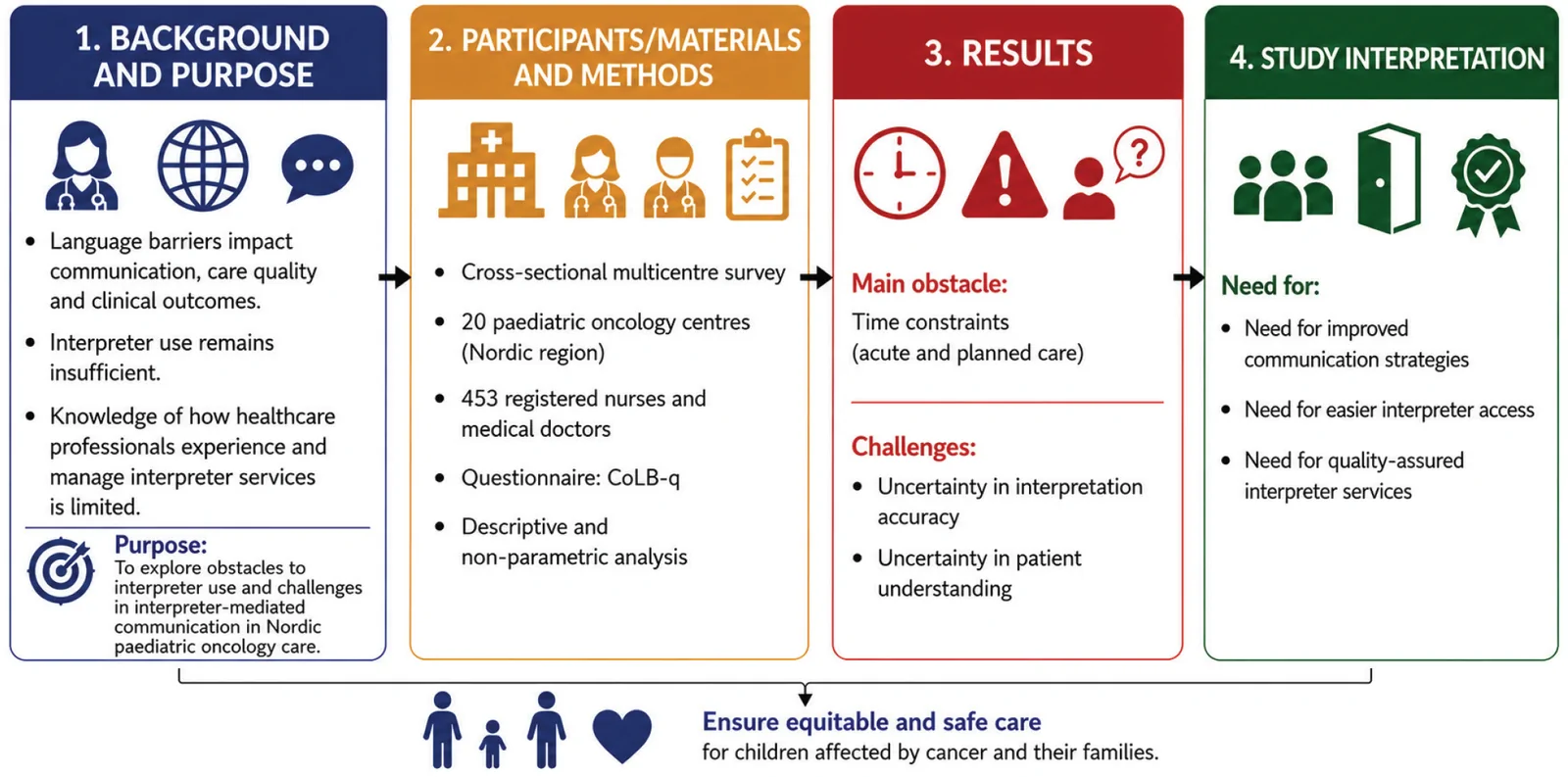

## Introduction

Having a seriously ill child is stressful and even more so when parents and healthcare professionals (HCPs) do not speak the same language [1]. Limited proficiency in the majority language (LPML) can hinder parents’ understanding of their child’s medical condition and their ability to communicate the child’s symptoms and healthcare needs. This can lead to considerable helplessness in an already vulnerable situation, as well as fear that language-related misunderstandings can affect the child’s care [1, 2]. Research have consistently demonstrated that many of these difficulties can be mitigated through the use of interpreter services by HCPs when caring for families with LPML, thereby promoting safer and more equitable care [1–5].

In paediatric oncology, communication can be troublesome even for families who speak the majority language, due to the complexity of the medical information and potentially life-threatening condition [6–9]. The cancer diagnosis involves a long and information‑heavy process, requiring prolonged, and varied communication throughout the entire course of care [9]. Families with LPML risk misunderstandings, reduced access to information and resources, and decreased parental confidence and advocacy, all of which may affect the quality of care [2]. Despite having access to the same paediatric oncology care, children with a foreign background risks a higher mortality [10]. According to Kyrönlahti et al. [10] language barriers may contribute to this disparity and highlights a need for tailored, language‑adapted information for families from diverse backgrounds to ensure equitable access to paediatric oncology care [10]. Furthermore, language barriers negatively influence paediatric cancer care outcomes [11].

All Nordic countries offer publicly funded, universal healthcare, but the legislation of interpreter services varies. In Denmark, interpreter services are subject to a fee after 3 years of residence for adults seeking healthcare [12]. In Norway, it is prohibited to use children as language brokers in interactions with authorities [13]. There are persistent gaps in interpreter provision in paediatric care in the Nordic countries, leading to delays in for example discharge processes [14].

Approximately 30% of children (0–17 years old) in Sweden have at least one foreign born parent [15]. The statistics suggests it is a potentially large proportion of families who might require interpreters when accessing healthcare services. The Health and Medical Care Act [16] form the foundation for the work carried out by HCPs, ensuring good health and care on equal terms. A core aspect is that families receive adequate information about their child’s health and treatment. The Swedish Patient Act [17] requires that information must be adapted to the patient’s linguistic background and that the person providing the information should ensure that patients and families have understood both the content and the significance of the information provided. This makes it the responsibility of HCPs to facilitate effective communication, including the appropriate use of interpreters to uphold these rights.

While legislation affirms families’ right to tailored information, research indicates a significant underuse of interpreters [11, 14], leading to a compromised quality of care, patient safety and family involvement in care [2–5, 18]. Interpreter use is associated with higher patient satisfaction, better clinical outcomes, shorter hospital stays, and fewer medical errors [18–20]. However, interpreter use is justified by more than their advantages; when interpreters are not used, children are often relied upon as language brokers [3, 5]. This can negatively affect children, particularly when they are exposed to inappropriate or sensitive information, placing an undue responsibility on them in matters related to their own care [21].

Language barriers add an additional layer of vulnerability to an already highly stressful situation for children and families in paediatric oncology care. When interpreters are not used appropriately, language barriers lead to disparities in care and pose a threat to patient safety. Although, the use of interpreters is essential for ensuring high-quality, safe and equitable care, interpreters remain persistently underused in healthcare. Knowledge of how HCPs experience and manage interpreter use is limited, yet essential for understanding how it can be improved. The aim of the study was to explore HCPs’ experiences of obstacles to interpreter use in Nordic paediatric oncology care, and challenges in interpreter-mediated conversations. For the purpose of this study, obstacles were understood as factors that prevented or limited the use of interpreter services by HCPs, whereas challenges were understood as difficulties that arose during the communication process when an interpreter was involved.

## Participants/materials and methods

### Design, setting and participants

This multicentre, cross-sectional survey included all Nordic paediatric oncology centres: Sweden (*n* = 6), Finland (*n* = 5), Denmark (*n* = 4), Iceland (*n* = 1) and Norway (*n* = 4). Paediatric cancer treatment in these countries is centralised to paediatric oncology centres at public university hospitals, which coordinate care in collaboration with shared care hospitals. The study size was determined by the number of eligible centres and the number of registered nurses (RNs) and medical doctors (MDs) working in those centres. All RNs, nursing assistants and MDs involved in direct patient care in inpatient and outpatient settings were invited to participate. As 96% of the nursing assistants originated from the Swedish cohort, this group was excluded from the analyses to avoid an overrepresentation of Swedish participants within one professional category. This decision was made to enhance the comparability between cohorts and to reduce the potential influence of country-specific confounding factors. The STROBE checklist for observational studies was followed.

### Questionnaire

The Communication over Language Barriers questionnaire (CoLB-q) [22] was used to assess HCPs’ experiences of communicating across language barriers and the use of interpreters in paediatric care. Originally developed in Swedish, the CoLB-q was translated into Finnish, Danish, Icelandic, and Norwegian. Separate translations were conducted by an informed HCP, fluent in the majority language and Swedish, and a professional translator. The two versions were compared and synthesised in focus group of HCPs fluent in the majority language of the respective countries. The questionnaire consists of 17 questions: 14 closed and three open-ended questions. The questions concerning obstacles to interpreter use were defined in this study as factors that hinder the use of interpreters, such as time constraints in acute and routine care situations, and financial restrictions. Other questions concerned challenges in interpreter-mediated communication, which were defined as difficulties arising during interpreter encounters, including not knowing whether the patient has received the correct information, or what the patient has understood of the conversation. Some questions present dichotomous answer choices (Yes or No) while some use a four-point Likert type scale (never, seldom, sometimes and often). One question presents alternatives: not at all, to a low degree, not so high degree and to a high degree. The questionnaire also includes 10 demographic questions covering profession, gender, education level, workplace, and length of work experience in paediatric care. Participants were instructed to answer the questionnaire based on their experiences in their current work setting. Those who worked across multiple units were asked to consider the unit where they spent most of their working time. As the survey captures multiple aspects of interpreter use and communication across language barriers, different sections of the questionnaire have been analysed and reported separately according to distinct study aims and research questions. Demographic data and responses to the first set of items addressing general communication experiences have been published [23]. The present study focuses exclusively on the remaining, unpublished items concerning obstacles and challenges to interpreter use. Therefore, participants who reported not using interpreters were excluded, as the focus was to explore such factors among those with actual experience of interpreter use.

### Data collection

Data collection was conducted between 2016 and 2022. The study was initiated in Sweden 2016, followed by Finland and Denmark in 2019, Iceland in 2021, and ended in Norway 2022. Data collection was performed by research nurses, consultant RNs and members of the working group on ethics of the Nordic Society of Paediatric Haematology (NOPHO). In Sweden, Iceland, and Finland, paper questionnaires were distributed and collected by local coordinators and returned by post. In Denmark and Norway, web-based questionnaires were administered, with private links sent by the research team or a public link sent by local study coordinators to the HCPs’ professional email addresses. The survey was confidential as the research team had access only to the provided answers, including demographic information.

### Statistics

Given the aim of the study, only descriptive and bivariate analyses were performed. Descriptive statistics were used to summarise the data and are presented as frequencies and percentages (*n*, %). Associations between categorical variables were evaluated using Pearson’s chi-square test. When the expected cell count was < 5 in more than 20% of the cells, the Fisher–Freeman–Halton exact test was applied. Group comparisons were performed between professions and between countries. A *p*-value < 0.05 was considered statistically significant. No replacement or estimation of missing data were done. For each survey item, results are presented using only the participants who answered that specific question.

## Results

A total of 881 RNs and MDs, working in paediatric oncology centres in the Nordic countries, were invited to participate, and 489 returned the survey, yielding an initial response rate of 55%. Respondents were excluded if they did not work in direct patient care (*n* = 12), did not answer the question about working in direct patient care (*n* = 2), had not answered any survey questions (*n* = 6), or reported that they never communicated with the help of an interpreter (*n* = 16). After exclusions, 453 responses remained: 362 RNs and 91 MDs.

### Participants

Most participants had more than 5 years of experience in paediatric oncology care and a completed continued education. A majority worked in inpatient care, and about half reported often caring for patients and families with LPML. Overall, 19% had received education or training in using interpreters, which was most common in Norway. Demographic characteristics are presented in Table 1, while Supplemental Table 1 shows interpreter training by country and profession.

**Table 1:** Demographic and professional characteristics of participants.

Characteristic	All participants, *n* (%)
**Profession** (*n* = 453)	
Registered nurse	362 (80)
Medical doctor	91 (20)
**Experience in paediatrics** (*n* = 451)	
≥ 5 years	324 (72)
< 5 years	127 (28)
Continued education* (*n* = 447)	
Yes	270 (60)
No	177 (40)
**Work setting** (*n* = 450)	
Inpatient care	282 (63)
Outpatient care	31 (7)
Both inpatient and outpatient care	137 (30)
**Country** (*n* = 453)	
Denmark	74 (16)
Finland	93 (21)
Iceland	21 (5)
Norway	69 (15)
Sweden	196 (43)
**Frequency of interpreter use in the following ways**	
**Interpreter on-site** (*n* = 449)	
Never	5 (1)
Seldom	86 (19)
Sometimes	225 (50)
Often	133 (30)
**Interpreter via telephone** (*n* = 450)	
Never	38 (8)
Seldom	125 (28)
Sometimes	211 (47)
Often	76 (17)
Interpreter via video** (*n* = 435)	
Never	343 (79)
Seldom	74 (17)
Sometimes	16 (4)
Often	2 (1)

Note. Percentages are based on available responses for each variable; totals and the number of respondents may vary due to missing data.

* Including specialist or professional training.

** Minor discrepancies in percentage totals are due to rounding.

### Coordination of HCPs

Coordinating healthcare interventions and professions when an interpreter was booked on-site was commonly reported, with 38% reporting that it occurred sometimes and 45% reporting often. However, country-level differences were observed: in Sweden and Finland, over 60% reported coordinating care often, whereas in Norway and Denmark, it was more common to report sometimes. This variation was statistically significant (*p* < 0.001) (Table 2). RNs and MDs reported this to a similar extent.

**Table 2:** Answers to the question: how often do you coordinate several health interventions or professions when the interpreters are present?

Response options	Never *n* (%)	Seldom *n* (%)	Sometimes *n* (%)	Often *n* (%)	*p*
**All participants** (*n* = 449)	15 (3)	62 (14)	170 (38)	202 (45)	
**Denmark** (*n* = 74)	3 (4)	23 (31)	40 (54)	8 (11)	
**Finland** (*n* = 92)*	2 (2)	3 (3)	28 (30)	59 (64)	
**Iceland** (*n* = 21)	1 (5)	3 (14)	9 (43)	8 (38)	
**Norway** (*n* = 68)	4 (6)	19 (28)	41 (60)	4 (6)	
**Sweden** (*n* = 194)	5 (3)	14 (7)	52 (27)	123 (63)	
**Overall** *p*-value**					< 0.001

Note. Percentages are based on available responses for each variable; totals and the number of respondents may vary due to missing data.

* Minor discrepancies in percentage totals are due to rounding.

** Fisher–Freeman–Halton exact test comparing all countries.

### Obstacles to using interpreters

The distribution of reported obstacles is presented in Table 3. Among all respondents, lack of time in acute care situations was the most reported obstacle: 42% reported it occurred sometimes and 23% often. In planned routine care situations, lack of time was reported more rarely as an obstacle. Economic restrictions were uncommon, with 81% reporting never.

**Table 3:** Answers to the question: how often do you experience any of the following as obstacles to the use of interpreters?

Response options	Never *n* (%)	Seldom *n* (%)	Sometimes *n* (%)	Often *n* (%)
**Economic restrictions** (*n* = 435)	352 (81)	53 (12)	23 (5)	7 (2)
**Lack of time in acute care situations** (*n* = 442)	48 (11)	107 (24)	186 (42)	101 (23)
**Lack of time in planned routine care situations** (*n* = 442)	74 (17)	167 (38)	165 (37)	36 (8)

Note. Percentages are based on available responses for each variable; totals and the number of respondents may vary due to missing data.

**Figure 1:**
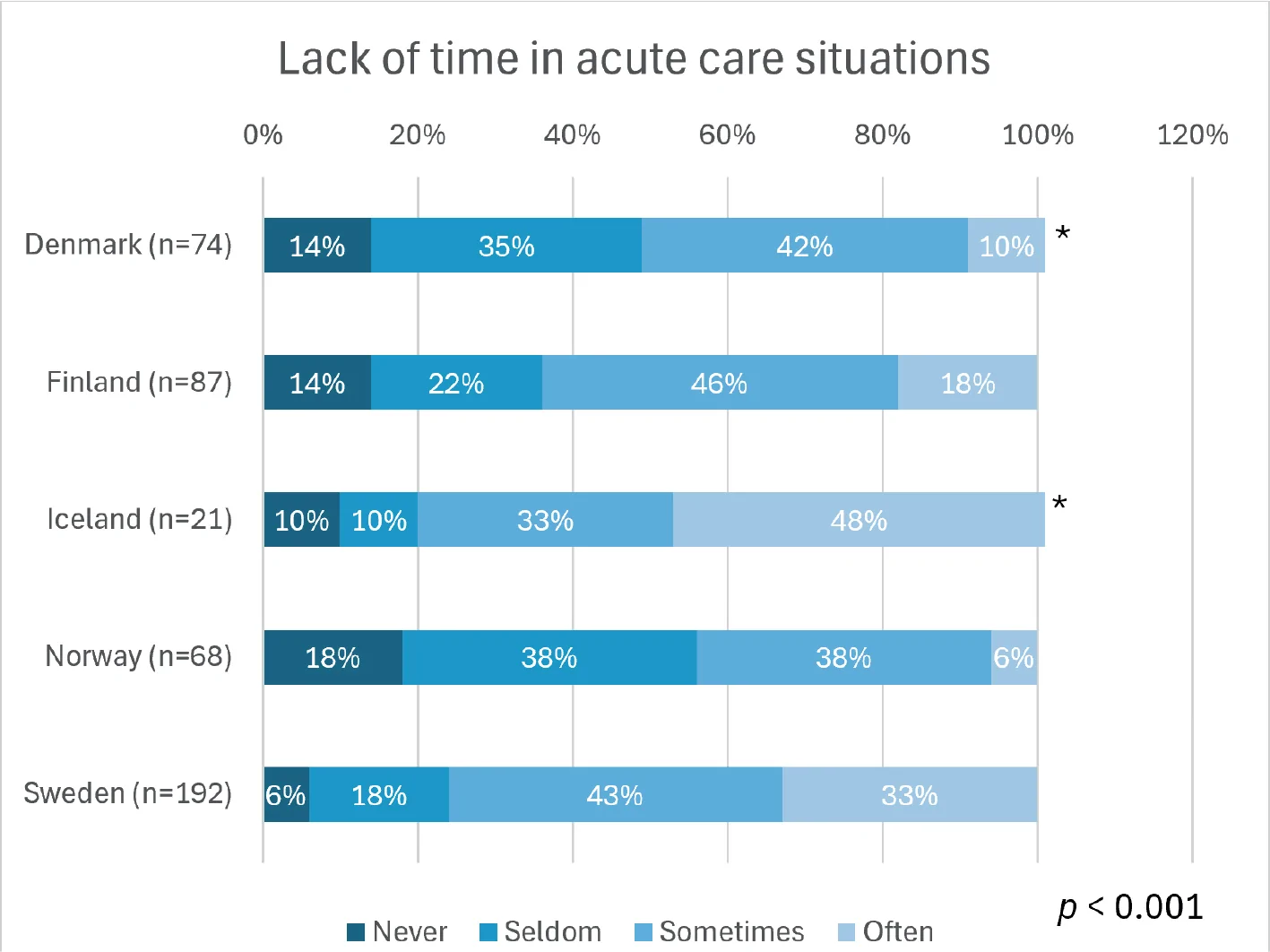
Distribution of responses regarding lack of time in acute care situations, *n* = 442. Note. Percentages are based on available responses for each variable; totals and the number of respondents may vary due to missing data. *Minor discrepancies in percentage totals are due to rounding.

It was most common in Iceland that lack of time in acute situations was experienced as an obstacle to interpreter use: 48% reported it often, while in Norway and Denmark less than 10% reported likewise (*p* < 0.001) (Figure 1). Although financial restrictions were uncommon, there were statistically significant differences between countries (*p* < 0.001). In Norway, no respondents reported economic restrictions being often an obstacle, and few did so in Sweden, Denmark, and Finland, whereas 10% in Iceland reported often.

**Figure 2:**
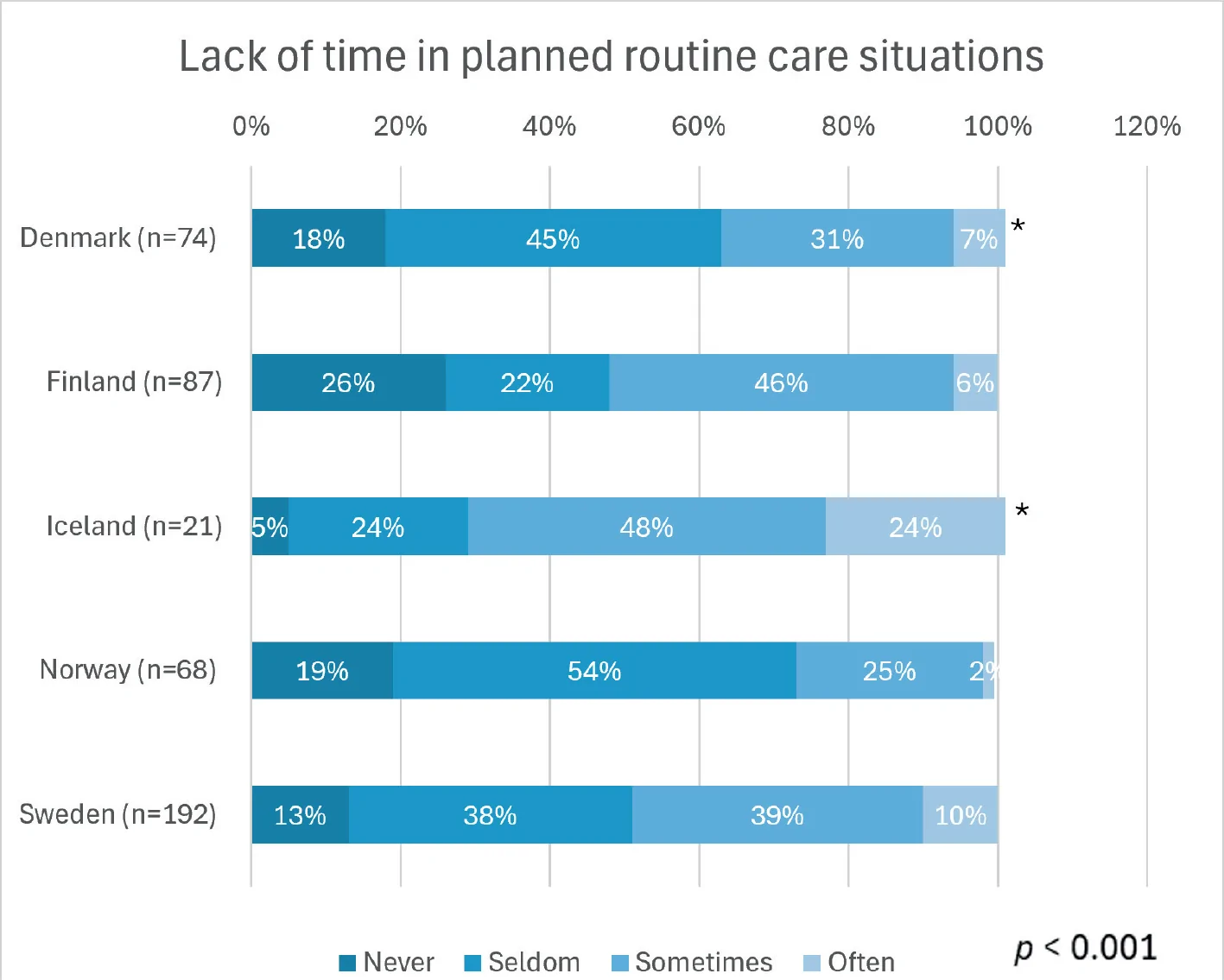
Distribution of responses regarding lack of time in planned routine care situations, *n* = 442. Note. Percentages are based on available responses for each variable; totals and the number of respondents may vary due to missing data. *Minor discrepancies in percentage totals are due to rounding.

Moreover, in planned routine care situations, at least 25% of respondents in all countries reported that lack of time was sometimes an obstacle to using an interpreter. However, there were statistically significant differences between countries (*p* < 0.001) (Figure 2). At the same time, around 20% of respondents in Denmark, Norway, and Finland reported that lack of time in routine care was never an obstacle, while in Sweden 13% reported never (Figure 2). Although, no significant differences were found between professions.

### Challenges during the interpreted-mediated conversation

Overall, the most reported challenge during interpreter-mediated conversations was controlling what the patient had understood from the conversation (Table 4). Most respondents reported that they sometimes felt uncertain whether the patient had received correct information. Being left out of the conversation when the interpreter and the patient/family spoke with each other was less common, though nearly one-third reported that they sometimes had experienced it (Table 4).

**Table 4:** How often have you experienced the following challenges in the interpreter-mediated conversation?

Response options	Never *n* (%)	Seldom *n* (%)	Sometimes *n* (%)	Often *n* (%)
**Uncertain whether the patient has received the correct information** (*n* = 443)	15 (3)	141 (32)	238 (54)	49 (11)
Trouble controlling what the patient understood of the conversation* (*n* = 441)	11 (3)	105 (24)	247 (56)	78 (18)
**Left out of the conversation** (*n* = 441)	86 (20)	204 (46)	124 (28)	27 (6)
**Time reserved for the conversation was insufficient** (*n* = 443)	40 (9)	185 (42)	168 (38)	50 (11)

Note. Percentages are based on available responses for each variable; totals and the number of respondents may vary due to missing data.

* Minor discrepancies in percentage totals are due to rounding.

**Figure 3:**
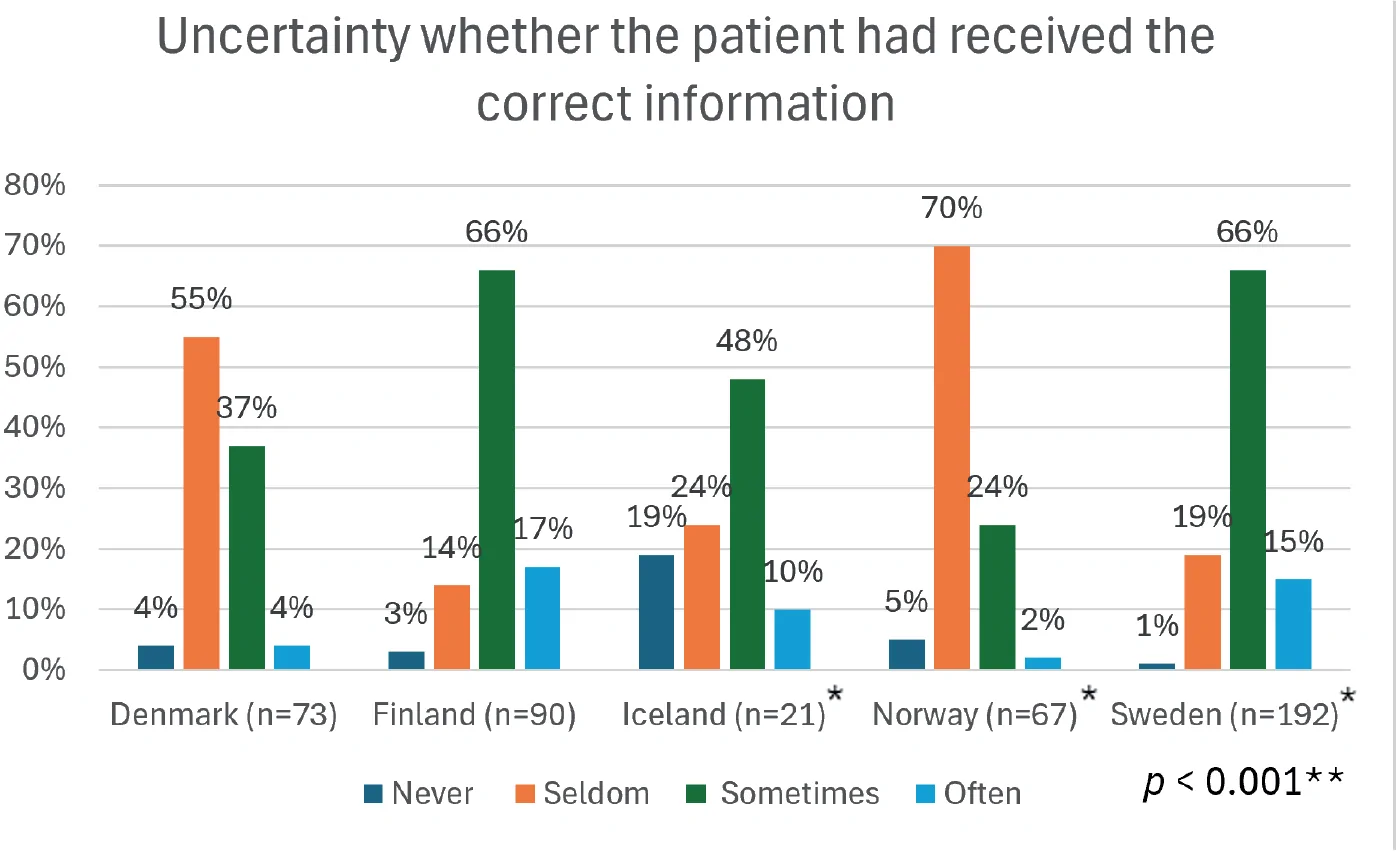
Distribution of responses by country regarding uncertainty whether the patient had received the correct information during the interpreter-mediated conversation, *n* = 443. Note. Percentages are based on available responses for each variable; totals and the number of respondents may vary due to missing data. *Minor discrepancies in percentage totals are due to rounding. **Fisher–Freeman–Halton exact test comparing all countries.

When comparing countries, uncertainty whether the patient had received correct information was more common in Sweden and Finland, with over half of the respondents reporting sometimes, while in Norway, 70% reported seldom (*p* < 0.001) (Figure 3). Differences between countries were also evident in the extent to which respondents reported difficulties in controlling what the patient had understood from the conversation. In Sweden and Finland, about twice as many respondents reported this often being troublesome compared to the other Nordic countries (*p* < 0.001).

None or few respondents (between 0 and 14%) reported often feeling left out of the conversation. Over 25% reported that the time reserved for the interpreted-mediated conversation was sometimes insufficient. This was most common in Sweden and Finland, where a higher proportion reported that the time reserved was often insufficient.

When comparing professions, 17% of MDs and 10% of RNs reported often feeling uncertain whether the patient had received correct information, with no significant differences between the professions. Most respondents in both groups found it sometimes challenging to assess what the patient had understood from the conversation. MDs were more likely to report feeling left out of the conversation, with 38% reporting sometimes, compared to 25% of the RNs, although this difference was not significant.

### Ensuring patient understanding during interpreter-mediated conversations

To ensure that the family/patient had understood the given information, the respondents reported that they sometimes (39%) or often (52%) asked the patient or their family via the interpreter if they had understood the given information. It was less common to ask patients or family members to explain in their own words what they had understood via the interpreter (Supplementary Table 2).

When comparing countries, some differences were found in checking comprehension. In Sweden and Finland, 69% vs 62% reported that they often asked via interpreter if the family had understood the given information, while in the other Nordic countries, it was more common to do it sometimes. Furthermore, it was more common in Sweden (22%), Finland (21%), and Denmark (20%) to often ask patients/families to explain in their own words what they had understood, compared to 10% in Iceland and Norway.

When comparing professions, most MDs and RNs reported they either sometimes or often asked the patient or family via the interpreter if they had understood the given information. This practice was more common among MDs where 64% reported often, compared to 49% among the RNs. Over 30% in each professions reported that they sometimes asked patients or family members to explain in their own words what they had understood via the interpreter. The proportion of MDs (36%) who reported doing this often was twice as high as that of RNs (15%), and this was a statistically significant difference (*p* < 0.001).

## Discussion

For this study, obstacles were understood as factors hindering the use of interpreters, and challenges as difficulties arising during interpreter-mediated communication. The findings illuminate the central obstacles and challenges that shape interpreter use in clinical practice. Time constraints emerged as the predominant obstacle, underscoring how structural and organisational pressures can directly limit the feasibility of engaging interpreters even when clinically warranted. Equally significant, the most frequently reported challenge concerned HCPs’ difficulties in accurately gauging patients’ understanding during interpreter-mediated encounters.

Lack of time was the most frequently reported obstacle to interpreter use, particularly in acute care situations. This gives cause to concern as urgent situations require rapid and accurate communication. Previous studies also identify time as an obstacle for using interpreters, often due to the time required to arrange interpreter services or the longer duration of interpreter-mediated conversations [24–28]. The findings highlight a need for organisations to enable interpretation despite time pressure, by implementing easier access to interpreters or alternative solutions, i.e. digital interpreter services. To meet the demands of safe care even in stressful situations and to accommodate urgent requests, a plausible strategy could be improving the quality of digital interpreter services [25, 27, 29, 30]. In addition, better organisation of interpreter services may facilitate use and help address time constraints [25–28].

In our study, many HCPs reported challenges in assessing whether patients had understood the information conveyed via interpreters. This aligns with other studies where HCPs report concerns regarding the accuracy of the transmission of medical information [27, 28, 31]. Difficulties in assessing whether patients have understood information should be taken seriously, given the complexity of information in paediatric oncology [7, 8], risking patient safety and family involvement [1–4]. While the present study did not evaluate strategies to improve communication, other studies suggest that targeted education and training in the practical use of interpreters for HCP improves communication and understanding between providers, interpreters, and patients [26–30, 32]. Such education could provide HCPs with competencies required to effectively conduct interpreter-mediated communication in healthcare, strengthen communication accuracy, and promote patient-safe care.

Although many respondents in our study frequently asked patients/families about their understanding, it was less common to confirm it by asking them to explain in their own words. Asking patients/families to restate what they have understood can enhance their safety and involvement in care [33]. Moreover, repetition of information is essential even when the families are proficient in the local language, supporting their understanding and processing of information in paediatric oncology care [9].

Our study found differences between countries. Time and financial obstacles were most frequent in Iceland, while in Norway, fewest challenges with interpreter accuracy and patient understanding were reported. Even time constraints were least common in Norway. Although these differences should be interpreted cautiously due to variations in sample sizes across the countries, and may reflect variations in national guidelines and organisational structures. In 2022, the Norwegian Interpreting Act entered into force, requiring public bodies to use qualified interpreters and prohibiting the use of children as language brokers [13]. In Sweden, the right to an interpreter is regulated in several laws but framed primarily as a patient right rather than a legal duty, while in Norway, legislation places a more explicit obligation on public bodies to use qualified interpreters [13]. Norway also has a national system for quality assurance of interpreters [34], enabling hospitals to easily book qualified interpreters through a centralised register. Although Sweden has a similar system, there are little incentives for interpreters to get education, and to pass the state authorisation. Consequently, there are fewer qualified medical interpreters in Sweden, which leads to uneven quality. While legislative differences may be a plausible explanation for how interpreter services are organised and quality-assured, this interpretation should be considered as tentative.

Finally, our results showed that coordination of care by sharing an interpreter with other HCPs when interpreters were available, was common in all countries. This can be efficient, coordinating several HCPs around one interpreter encounter, but may lead to compact information [35], and complicate the adaptation of information to the needs of the family [24, 36]. This highlights the need to improve the organisation and planning of interpreting services. It also underscores the importance of adopting a longer-term perspective on children’s care and their interactions with healthcare.

### Strengths and limitations

The sample size (*n* = 453), including 20 Nordic paediatric cancer care centres, provides a broad understanding of obstacles and challenges to interpreter use. The data were collected using a validated instrument developed in the paediatric oncology context. Furthermore, most participants reported that they often cared for patients and families with LPML, indicating that they recurringly encounter situations requiring interpreters.

One limitation is that self-reported data mirror participants’ own perceptions. For example, the Likert-scale response options (‘never’, ‘seldom’, ‘sometimes’, and ‘often’) are not operationally defined in the CoLB-q. Consequently, participants may have interpreted these frequency categories differently based on their own subjective perceptions and experiences. Another important limitation is that the data collection was conducted over an extended period of time and was also affected by the COVID-19 pandemic. Furthermore, this means that the rapid digital development following the pandemic has likely influenced the way digital translation tools are used today and may affect the applicability of the results. The difference in the distribution of participants across countries and professions could be considered a limitation as it limits the ability to draw reliable conclusions about observed differences. This includes the exclusion of participants (*n* = 16) who reported never using interpreters, which may be considered a limitation, as some HCPs may refrain from using interpreters due to perceived obstacles and challenges. The study was conducted in paediatric oncology care, which may limit the generalisability of the findings. Furthermore, as the study is descriptive, no adjustment for multiple testing was performed. Consequently, *p*-values should be interpreted cautiously due to multiple testing and therefore risk for type 1 error.

### Study interpretation

This is the first study to explore HCPs’ experiences of interpreter use in paediatric oncology care across the Nordic countries. The results highlight a vulnerability in communication, as reliance on an intermediary can limit awareness of patient comprehension. The findings suggest that interpreter-mediated communication introduces additional challenges to an already complex clinical context, particularly when discussing sensitive information and assessing patient and family understanding. HCPs reported difficulties in determining whether information had been accurately conveyed and comprehended, highlighting the reliance on interpreters as a crucial link in the communication process. The results indicate that obstacles and challenges to interpreter use are not solely related to individual HCPs’ pedagogical communication skills but are also influenced by organisational and structural factors, such as interpreter availability, accessibility, and perceived quality of interpretation services. These findings suggest that improving interpreter-mediated communication requires interventions at multiple levels, including organisational support, easier access to qualified interpreters, and systems for monitoring and ensuring the quality of interpreter services. Addressing these challenges may contribute to more equitable, safe, and family-centred care for children with cancer and their families who have LPML in the Nordic countries.

## Supplementary Material





## Data Availability

Data will be made available upon reasonable request.
