# Supplementary material for: Interpreter use in Nordic paediatric oncology care – a multicentre cross-sectional study
Source: Acta Oncol. 2026 Sep 8;65:46078. doi: 10.2340/1651-226X.2026.46078 (PMC13575807; doi:10.2340/1651-226X.2026.46078)
Supplement: Supplementary file 2 [file AO-65-46078-s2.pdf]

Supplementary material has been published as submitted. It has not been copyedited, or typeset by Acta Oncologica

Supplemental material

Supplementary table 1: Proportion of participants reporting education/training in interpreter use, by country and profession.

Characteristic	n (%)
**Education/training in using interpreters in all Nordic countries** (n=451)	
Yes	86 (19)
No	365 (81)
**Distribution by each country***	
Denmark (n=73)	8 (11)
Finland (n=92)	18 (20)
Iceland (n=21)	3 (14)
Norway (n=69)	23 (33)
Sweden (n=196)	34 (17)
**Distribution by profession in all Nordic countries*** (n=451)	
Registered nurse (n=360)	61 (17)
Medical doctor (n=91)	25 (28)

Note. Percentages are based on available responses for each variable; totals and the number of respondents may vary due to missing data.

*Percentages are calculated within each country/profession denominator shown in parentheses.

Supplementary table 2: Answers to the question about how often the respondent use one of the following ways to ensure that the patient has understood the information that was given via the interpreter.

	**Never n (%)**	**Seldom n (%)**	**Sometimes n (%)**	**Often n (%)**
**Ask the patients/families (via the interpreter) if they have understood *** (n=442)	8 (2)	33 (8)	173 (39)	228 (52)
**Ask the patients/families to tell me by themselves (via the interpreter) what they have understood** (n=444)	32 (7)	123 (28)	204 (46)	85 (19)

Note. Percentages are based on available responses for each variable; totals and the number of respondents may vary due to missing data.

* Minor discrepancies in percentage totals are due to rounding.
